# Contribution of bacterial outer membrane vesicles to innate bacterial defense

**DOI:** 10.1186/1471-2180-11-258

**Published:** 2011-12-01

**Authors:** Andrew J Manning, Meta J Kuehn

**Affiliations:** 1 Department of Biochemistry, Duke University Medical Center, Box 3711, 307 Research Drive, Durham, NC 27710 USA

## Abstract

**Background:**

Outer membrane vesicles (OMVs) are constitutively produced by Gram-negative bacteria throughout growth and have proposed roles in virulence, inflammation, and the response to envelope stress. Here we investigate outer membrane vesiculation as a bacterial mechanism for immediate short-term protection against outer membrane acting stressors. Antimicrobial peptides as well as bacteriophage were used to examine the effectiveness of OMV protection.

**Results:**

We found that a hyper-vesiculating mutant of *Escherichia coli *survived treatment by antimicrobial peptides (AMPs) polymyxin B and colistin better than the wild-type. Supplementation of *E. coli *cultures with purified outer membrane vesicles provided substantial protection against AMPs, and AMPs significantly induced vesiculation. Vesicle-mediated protection and induction of vesiculation were also observed for a human pathogen, enterotoxigenic *E. coli *(ETEC), challenged with polymyxin B. When ETEC with was incubated with low concentrations of vesicles concomitant with polymyxin B treatment, bacterial survival increased immediately, and the culture gained resistance to polymyxin B. By contrast, high levels of vesicles also provided immediate protection but prevented acquisition of resistance. Co-incubation of T4 bacteriophage and OMVs showed fast, irreversible binding. The efficiency of T4 infection was significantly reduced by the formation of complexes with the OMVs.

**Conclusions:**

These data reveal a role for OMVs in contributing to innate bacterial defense by adsorption of antimicrobial peptides and bacteriophage. Given the increase in vesiculation in response to the antimicrobial peptides, and loss in efficiency of infection with the T4-OMV complex, we conclude that OMV production may be an important factor in neutralizing environmental agents that target the outer membrane of Gram-negative bacteria.

## Background

Bacteria, especially pathogenic bacteria, must deal with a very hostile environment on a nearly continuous basis. How pathogenic bacteria first respond to this environment and lethal environmental stressors is a key element in their survival. Based on their initial response, either the pathogen may succumb and die, or it can respond and live despite its hostile surroundings. Long-term adaptive bacterial responses to antimicrobials include well-characterized mechanisms of expressing an altered version of the antibiotic target, enzymes to degrade the antibiotic, and transporters to remove the antibiotic [1]. Here, we consider the time immediately after the first exposure to a threat and before activation of long-term adaptive resistance to stressors. Understanding how bacteria mount this initial defense against stresses is critical to understanding how bacteria respond to, and survive, hostile environments.

We propose that bacterial outer membrane vesicles (OMVs) and OMV production may play a key role in innate bacterial defense against environmental stressors that target the cell envelope, as well as sub-inhibitory concentrations of outer membrane targeting antibiotics. OMVs are spherical portions of bacterial envelope containing outer membrane protein and lipid as well as soluble material contained in the lumen or bound to the external surface [2,3]. The role of OMVs in intercellular transport and signaling by pathogenic bacteria has been the subject of numerous studies [3]. However, only a few reports investigated more generally beneficial roles for OMVs that would explain their development in non-pathogenic Gram-negative bacterial species. Of these, some have described a role for OMVs in countering environmental stress and stressors. For instance, one report demonstrated that OMVs are induced by and protect bacteria from toluene exposure [4], and others reported that OMVs contribute to the formation of biofilms which have a well-known role in bacterial resistance to harsh environments [5,6]. In addition, Grenier *et al *discovered that OMVs from *Porphyromonas gingivalis *could protect cells against chlorhexidine, as well as provide degradative enzymatic activities to neutralize the killing abilities of human serum [7,8]. Furthermore, mutations resulting in hyper-production of OMVs were found to be advantageous when *E. coli *was challenged with otherwise lethal environmental stresses, including antimicrobials and ethanol, a general denaturant [4,9].

Natural antibiotics are common antimicrobial stressors encountered by bacteria in the environment as well as during infection of a host. Antimicrobial peptides (AMPs) are a key human defense to bacterial infections, as well as a defense employed by other Gram-positive and Gram-negative bacteria [10,11]. Antimicrobial peptides have also been found in a growing variety of other host organisms, including mice, insects, and frogs [12-15]. Few, however, acknowledge the sub-inhibitory concentrations of these defensins that pathogens commonly encounter on the epithelial surfaces, or in the environment [10,16]. The most common mechanism of action for these AMPs is alteration of bacterial membrane permeability, typically by pore formation [15,17,18]. Because of their generic target and their speed of action, AMPs have recently been revisited in the quest to develop novel antibiotics against Gram-positive and Gram-negative pathogens [14,19-22]. Currently, AMPs are used as a last line of defense against some multi-drug resistant pathogens [22-24]. Most bacterial AMP-resistance is characterized by lipid modifications to alter the charge of the outer membrane [25-27]. However these resistance pathways cannot fully explain the extent of resistance seen in Gram-negative bacteria [16]. We hypothesize that OMVs may act as a modulating intrinsic defense against AMPs as well as other outer membrane acting stressors, and that this defense may help to explain the gap in our current understanding of how Gram-negative bacteria respond to these compounds.

Phage are ubiquitous viral particles found in all niches of life, and at an estimated 10^31 ^phage on the earth, they are the most prevalent organisms on the planet [28,29]. A majority of these viral particles are considered to be bacteriophage, phage that specifically infect bacteria [28]. With the advent of metagenomics and the drive to study the microbiomes of not only environmental niches but also human niches, more and more bacteriophage are being discovered [30]. The addition of another player in the bacterial-host interaction matrix increases the complexity of the environment beyond what is currently appreciated, presenting yet another set of interactions to consider. Bacteriophage are specific to the host they replicate within [29]. Phage that infect Gram-negative bacteria typically identify their host by binding the outer membrane or one of its components [28]. As OMVs consist of components of the Gram-negative outer membrane, it seems logical that these blebs may play an important role in the interaction between bacteria and phage. Early work done by Loeb *et al *has already demonstrated a dramatic increase in outer membrane production and release in the presence of T4 phage in *E. coli *[31]. This study aims to characterize the interaction between OMV and T4 phage and determine its effect on the efficiency of phage infection.

In this work, we investigate the ability of OMVs to adsorb diverse outer membrane antimicrobial agents (AMPs and bacteriophage T4), and we determine if OMVs can contribute to the protection of Gram-negative bacteria against these lethal stressors. We examine if OMVs are induced in the presence of AMPs and investigate whether OMV-mediated protection and induction properties hold true for the human pathogen, enterotoxigenic *E. coli *(ETEC). We also investigate whether the presence of OMVs affect the ability of ETEC to express long-term, adaptive resistance to polymyxin B and the ability of *E. coli *to protect against phage over several replication cycles. Overall, our data support a model of intrinsic bacterial defense based on OMVs. This work supports the hypothesis that in certain environmental conditions, Gram-negative bacteria can use vesiculation as an immediate protective response.

## Results

### Increased survival by a hyper-vesiculating mutant after antimicrobial peptide stress

We first examined whether mutations that result in hyper-vesiculation protect bacteria against antimicrobial challenge. A wild-type (WT) laboratory *E. coli *and the isogenic hyper-vesiculating *yieM *mutant (Δ*yieM*) were selected for these studies. Compared to WT, a mutant harboring a transposon disruption of *yieM *hyper-vesiculates approximately 10-fold yet displays WT membrane integrity [9]. The full *yieM *knockout, Δ*yieM*, maintains all of the phenotypes previously described for the transposon mutant.

**Figure 1:**
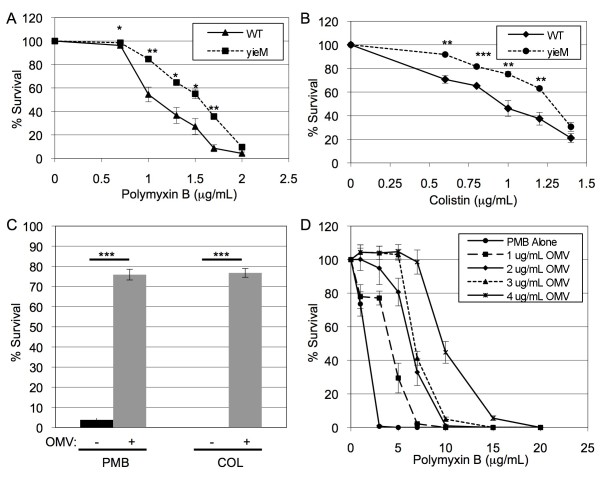
**OMV-mediated protection to AMPs**. Relative survival of WT (solid line) and Δ*yieM *(dashed line) *E.coli *after 2 h treatment with the indicated concentrations of polymyxin B (**A**) and colistin (**B**). (**C**) Cultures of mid-log phase WT *E. coli *were simultaneously treated with the indicated antibiotic (polymyxin B (PMB) 1.5 μg/mL and colistin (COL) 1.0 μg/mL) and either no OMVs (black bars) or with OMVs purified from WT *E.coli *(4 μg/mL) (grey bars). (**D**) To titrate OMV-mediated protection, indicated concentrations of WT OMVs were co-incubated in media for 2 h with indicated concentrations of polymyxin B and the media cleared of OMVs by centrifugation. Polymyxin B activity remaining in the media was assessed by adding the pretreated media to a mid log-phase culture of WT *E. coli*, incubating for 2 h, and plating for CFU. Relative growth (% Survival) was determined by dividing the CFU/mL obtained from antibiotic-treated cultures by the CFU/mL from cultures without antibiotic. (n = 9 for all experiments).

Polymyxin B and colistin are cyclic cationic antimicrobial peptides (AMPs) that act at the outer leaflet of the outer membrane, forming pores and altering membrane permeability [16,17,32]. Log-phase cultures of the strains were treated with the AMPs at a range of antibiotic concentrations just below the dose conferring ~10% survival of WT cultures. Cultures of the Δ*yieM *grew significantly better than WT in polymyxin B and colistin over a range of treatment doses (Figure 1A, B). Since the deletion of *yieM *does not cause a change in the lipid A structure of the LPS (Additional File 1, Figure S1B, C), these data suggest that hyper-vesiculation is protective against these AMPs. When treated with antibiotics that target peptidoglycan synthesis and protein synthesis (ceftriaxone, ampicillin, and tetracycline), the mutant demonstrated minimal or no change in growth phenotypes compared to the WT (data not shown). Together, these results suggest that vesicles can serve a protective function for some antibiotics, notably those antibiotics that interact significantly with the outer membrane.

### Outer membrane vesicles mediate protection against antimicrobial peptides

Secreted OMVs might help to defend cells against outer membrane-acting antibiotics based on the nearly identical surface constituents of the OMVs and the bacterial outer membrane. To address this possibility, we tested directly whether addition of purified OMVs could increase the survival of WT bacteria challenged with antibiotic. WT cultures were treated with antibiotic in the presence or absence of purified OMVs and growth was measured. The time course of the experiment was kept brief so the amount of OMVs the strain itself produced during the trial would be negligible compared with the quantity of OMVs added. A high OMV concentration was used in these initial experiments in order to detect whether there would be any effect. The simultaneous addition of OMVs with the polymyxin B or colistin treatment resulted in significantly increased survival compared to cultures treated with those antibiotics alone (Figure 1C). OMVs were not effective in protecting against ampicillin, ceftriaxone, or tetracycline, and OMVs alone had no effect on growth of untreated cultures (data not shown and Additional File 2, Figure S2).

To determine the extent by which OMVs could titrate AMP activity in the media, we incubated media containing a range of polymyxin B concentrations (0 to 20 μg/mL) with a range of OMV concentrations (0 to 4 μg/mL). The OMVs were removed via centrifugation and the polymyxin B activity remaining in the pre-incubated media was assessed indirectly. WT log-phase *E. coli *was treated with the pre-incubated media and bacteria survival was quantitated. We observed a depletion of polymyxin B activity in the media that depended on the concentration of OMVs (Figure 1D). The minimum ratio of OMVs to polymyxin B in the pre-incubation that resulted in complete protection was 4 μg OMV to 7 μg polymyxin B in 1 mL of culture. These data demonstrate that full mitigation of the bactericidal effects of polymyxin B could be achieved by the OMVs.

### Antimicrobial peptide treatment induces vesicle production

**Figure 2:**
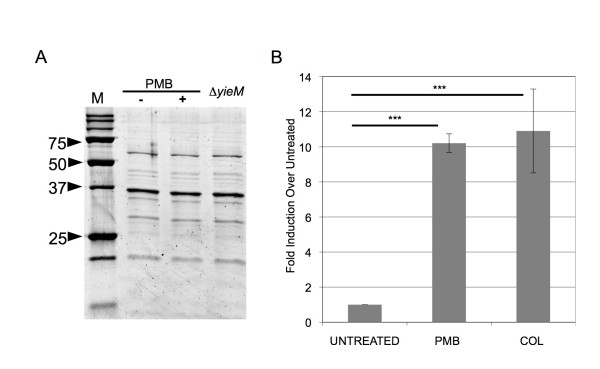
**OMV production is substantially induced by AMPs**. (**A**) OMVs from 0.75 μg/mL polymyxin B-treated (+) and untreated (-) WT cultures were purified, separated by SDS-PAGE, and stained using SYPRO Ruby Red. OMVs from strain Δ*yieM *are also shown for comparison. No significant differences in protein content could be identified across all samples. Molecular weight standards are indicated in kDa (M). (**B**) OMVs in the cell-free culture supernatant of antibiotic-treated WT cultures (0.75 μg/mL polymyxin B, PMB; or 0.5 μg/mL colistin, COL) were quantitated by measuring outer membrane protein and compared with the quantity of OMVs produced by untreated cultures (Untreated). Production was normalized to CFU/mL of each culture at the time of OMV preparation, and relative fold-differences are shown. (n = 9 for all experiments).

As protection was dependent on OMV concentration, we considered whether WT bacteria could be induced by antibiotic treatment to produce increased amounts of OMVs. For these experiments, it was particularly important to thoroughly control for the possibility that the antibiotic treatments would lyse cells, since this would obscure quantitation of OMVs. Therefore, we examined bacterial integrity for cultures treated with the maximum concentration of antibiotic that resulted in the lowest amount of killing (0.75 μg/mL polymyxin B, ≥ 95% survival; colistin 0.5 μg/mL). To test for the loss of cell wall integrity, the presence in culture supernatants of the constitutively-expressed periplasmic enzyme alkaline phosphatase (AP) was monitored. We prepared cell- and OMV-free supernatants from treated and untreated cultures and measured AP activity in the supernatants and corresponding cell pellets. The ratio of AP in the OMV-free supernatant for the treatments used for subsequent vesiculation induction assays was not significantly affected by the treatments (Table 1). We also examined the morphology of polymyxin B-treated cells by electron microscopy and found that the treated cells and the OMVs prepared from the induced cultures did not appear ruptured or morphologically different from untreated samples (data not shown). Furthermore, OMV and subcellular fractionation protein profiles for both treated and untreated cultures of *E. coli *were nearly identical (Figure 2A, Additional File 3, Fig S3). Together, this set of control experiments demonstrated that the antibiotic treatments did not affect cell integrity and that measurements of induced OMVs in treated cultures were not inaccurate due to products of cell lysis.

**Table 1:** Integrity of antibiotic-treated bacteria

		WC **(ng/mL)**		OMV-free Supe **(ng/mL)**		AP Leakage ([AP]_supe_:[AP]_whole cell_)*^a^*	
Strain	Treatment*^b^*	UNT	TRE	UNT	TRE	Untreated	Treated
MK318	Polymyxin B (0.75 μg/mL)	8.270 ± 1.010	7.870 ± 0.970	1.290 ± 0.080	1.341 ± 0.121	0.160 ± 0.007	0.170 ± 0.006
	
	Colistin (0.5 μg/mL)	9.440 ± 0.230	8.87 ± 0.07	1.20 ± 0.010	1.260 ± 0.021	0.127 ± 0.003	0.121 ± 0.002

ETEC	Polymyxin B (3 μg/mL)	6.100 ± 0.440	6.07 ± 0.510	1.201 ± 0.030	1.22 ± 0.030	0.198 ± 0.009	0.204 ± 0.020

ADA600	Untreated	0.020 ± 0.011	ND	0.024 ± 0.013	ND	ND	ND

*^a^*RFU measurements of AP in the OMV-free culture supernatant (Supe) compared to AP in whole cell (WC) pellets, normalized to CFU/mL in the culture. No significant differences in AP leakage between untreated (UNT) and treated (TRE) cultures were observed (p > 0.05).

*^b^*Treatments were for 2 h at 37°C; final concentration of treatments are shown. (n = 9)

To investigate whether vesiculation was induced upon treatment, we used a previously designed quantitative assay to measure OMVs in the culture supernatant [9]. Whereas other antibiotic (tetracycline, ampicillin, and ceftriaxone) treatments each modestly increased vesiculation (2 to 4 fold, data not shown), polymyxin B and colistin each increased OMV production substantially (10-fold) (Figure 2B). Therefore, the greatest induction of vesiculation occurred in response to the same antibiotics, polymyxin B and colistin, for which OMVs mediate protection.

### Protection and induction of OMVs produced by pathogenic *E. coli*

**Figure 3:**
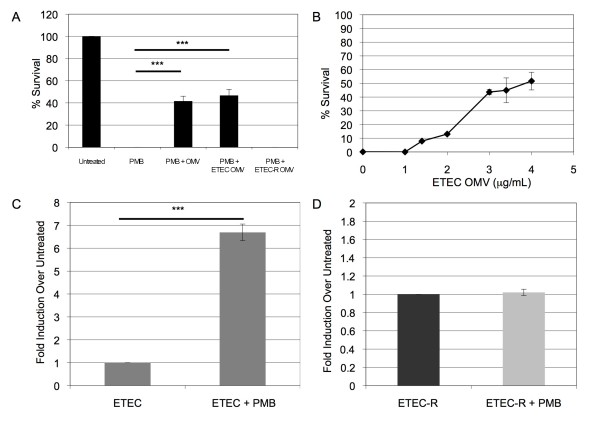
**ETEC, not ETEC-R, OMVs are protective and induced by polymyxin B**. (**A**) A mid-log culture of ETEC was treated with polymyxin B (4 μg/mL, final concentration) simultaneous with no addition (PMB), 10 μg of WT (K12) OMVs (PMB + OMV), 10 μg of ETEC-derived OMVs (PMB + ETEC OMV), or 10 μg ETEC-R-derived OMVs (PMB + R-ETEC-OMV). Relative growth (% Survival) was determined compared to cultures without antibiotic (Untreated). (n = 9) (**B**) To titrate OMV-mediated protection for ETEC, ETEC OMVs (final concentrations indicated) were added simultaneously with polymyxin B (5 μg/mL, final concentration) to a mid-log phase ETEC culture and co-incubated 2 h at 37°C. Relative growth (% Survival) was determined compared to cultures without antibiotic. (n = 6) OMV yield was quantitated for mid-log phase cultures of ETEC (**C**) or ETEC-R (**D**) treated for 14 h with 3 μg/ml polymyxin B. (n = 6 for both **C **and **D**) OMV production was normalized to the CFU/mL of each culture at the time of vesicle harvest, and relative fold-differences compared to untreated cultures are shown.

We studied a clinical isolate of enterotoxigenic strain of *E. coli *(ETEC) to evaluate whether OMV-mediated protection and stress-induced OMV production also occurs for a pathogenic strain of *E. coli*. Although this ETEC strain is intrinsically more resistant to polymyxin than K12 *E. coli*, the addition of either purified K12 OMVs or ETEC OMVs to ETEC cultures further protected the bacteria from killing by polymyxin B (Figure 3A). By titrating in purified ETEC OMVs, we observed that the survival of a mid-log phase culture of ETEC treated with 4 μg/mL polymyxin significantly increased from 0% to nearly 50% with the addition of 3-4 μg/mL ETEC OMVs (Figure 3B).

In addition, although ETEC already produces a higher basal level of OMVs than K12 strains, ETEC OMV production was significantly induced after polymyxin B treatment (nearly 7-fold) as compared to untreated cultures (Figure 3C). Control experiments confirmed that the treatment did not cause significant cell lysis (< 5% reduction of CFU and no significant change in periplasmic AP in the OMV-free culture supernatant, Table 1). Thus, upon AMP challenge, both K12 and pathogenic *E. coli *strains are induced to produce protective OMVs.

### OMV-mediated protection and induction of OMVs depend on the antibiotic sensitivity of the strain

We next considered the likelihood that OMVs adsorb polymyxin B by the interaction between OMV lipopolysaccharide (LPS) and the antibiotic. Based on the fact that polymyxin resistant strains produce modified LPS that cannot bind polymyxin B [27,33], we predicted that OMVs produced by a resistant strain would not interact with polymyxin B and, consequently, would not confer protection to a sensitive strain. To test this, we derived a polymyxin-resistant strain of ETEC (ETEC-R) by treating mid-log phase ETEC cultures with a high concentration of polymyxin B. LPS isolated from ETEC-R was analyzed by mass spectroscopy and was confirmed as having a modified lipid A consistent with a phosphoethanolamine attached to the phosphate in the 1 position (Additional File 1, Figure S1E). This is consistent with previously seen lipid A modifications that alter the charge of the outer membrane [34].

OMVs purified from ETEC-R (R-OMVs) were simultaneously added with polymyxin B to a non-resistant ETEC culture. The ETEC-R-OMVs offered no protection at a concentration where ETEC-OMVs were previously seen to be maximally protective (Figure 3A). These data demonstrated that polymyxin B adsorption by the LPS of the OMV is the likely mechanistic basis for OMV-mediated resistance.

Interestingly, when we investigated polymyxin-induced vesiculation for ETEC-R, we found that vesicle production by ETEC-R did not significantly increase upon treatment with 10 μg/mL polymyxin B (Figure 3D). Therefore, it appeared that the mechanism by which OMV production is induced depends on polymyxin interaction with cellular LPS.

### OMVs alter antibiotic resistance phenotype in ETEC

**Figure 4:**
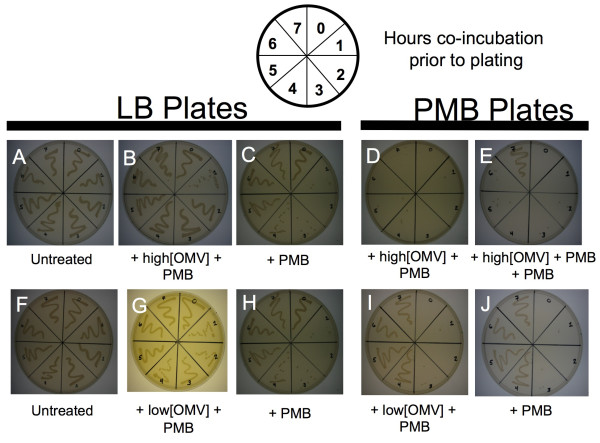
**Acquisition of ETEC resistance to polymyxin B is reduced by co-incubation with high concentrations of OMVs**. At hourly time-points for 0-7 h of co-incubation, equivalent volumes of the samples described below were streaked on each plate in a pattern indicated by the template diagram. *Top row: *ETEC co-incubated with (**A**) nothing, (**B**, **D**) a high concentration of ETEC OMV (2 μg/mL) and polymyxin B (3.5 μg/ml), or (**C**) polymyxin B alone (3.5 μg/mL). Samples were streaked either on LB agar (**A-C**), or LB containing 5 μg/ml polymyxin B (**D-E**). (**E**) ETEC co-incubated with ETEC OMV (3 μg/mL) and polymyxin B (3.5 μg/mL) for 5 h, then an additional 5 μg/mL polymyxin B was added, and plated on LB containing 5 μg/mL polymyxin B. Resistance was seen by hour 7 without decreasing cell population significantly. *Bottom row: *ETEC co-incubated with (**F**) nothing, or (**G**, **I**) 1.4 μg/mL ETEC OMV and 3.5 μg/ml polymyxin B, and (**H**, **J**) polymyxin B alone (3.5 μg/mL), streaked on LB (**F-H**) or LB containing 5 μg/mL polymyxin B (**I-J**). (n = 9 for all experiments).

Adaptive (longer-term) bacterial resistance to polymyxin is typically based on the upregulation of genes which lead to the modification of LPS [27,33]. We wondered whether OMV-mediated defense would affect the onset of adaptive resistance of ETEC to polymyxin B. A mid-log liquid culture of ETEC was treated with polymyxin B (3.5 μg/mL) and concurrently supplemented with either a relatively high concentration of ETEC OMVs (2 μg/mL) or buffer. Samples were taken hourly for up to 7 h post treatment, spread on LB agar and LB agar containing polymyxin B, and the plates inspected after 12 h incubation at 37°C (Figure 4). As expected from the results described earlier, ETEC cultures supplemented with OMVs survived better compared to cultures that did not contain added OMVs (Figure 4B, C). However, we further observed that these bacteria were not able to grow on plates containing polymyxin B (Figure 4D). This suggests that the bacteria survived to a greater extent but did not become adapted to resist polymyxin.

To test if the bacteria in the OMV-supplemented culture were simply incapable of becoming adaptively resistant, an additional 5 μg/ml polymyxin B was added at hour 5 after the OMV-polymyxin B co-incubation and the culture was then plated on polymyxin B-containing agar. Resistant ETEC were observed without a detectable decrease in cell number after 7 h (Figure 4E). This result demonstrated that the OMV-protected ETEC had the capacity to adapt to high levels of antibiotic and achieve resistance if the polymyxin dose was increased beyond the amount the OMVs could protect. This reasoning was confirmed in further experiments in which we used a lower OMV concentration (0.7 μg/ml) with the same concentration of polymyxin B. In these cultures, particularly at 1-4 h time points, fewer ETEC survived than for cultures where the higher OMV dose was added (Figure 4B, G), but the surviving bacteria more quickly adapted to polymyxin B, as demonstrated by their ability to grow on polymyxin B-containing agar (Figure 4I). When comparing the growth of supplemented and un-supplemented cultures, we conclude that low doses of OMVs promoted ETEC growth in polymyxin B at least 3 h earlier than with no added OMVs at all (Figure 4G, H). Thus, at low concentrations, OMVs confer immediate maintenance of bacterial viability and do not impede the activation of adaptive resistance. At higher concentrations, OMVs confer immediate resistance of the bacterial population but adversely affect bacterial acquisition of adaptive resistance.

### T4 Bacteriophage interact with OMVs and OMVs decrease efficiency of infection

**Figure 5:**
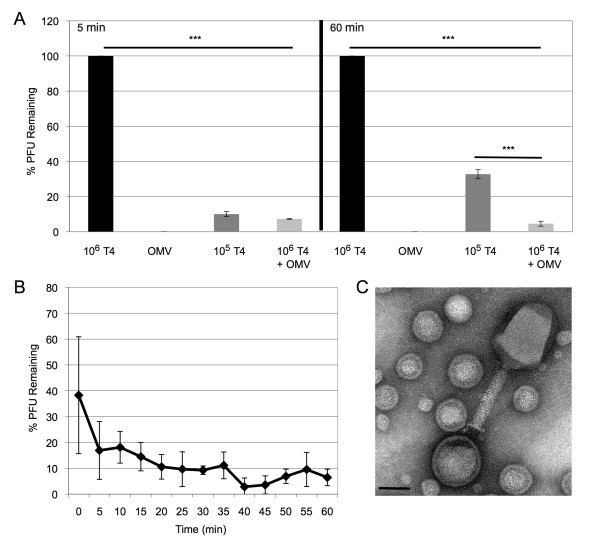
**T4 phage bind OMVs, reducing their capacity to infect *E. coli***. (**A**) 10^6 ^T4 phage were co-incubated with 1 μg purified WT OMVs (10^6 ^T4+OMV) for 2 h. As controls, 10^6 ^T4, 1 μg of purified WT OMVs, and 10^5 ^T4 were also incubated under the same conditions for 2 h. For the 5 min panel, samples were mixed with MK496 cells and allowed to incubate for 5 min, PFU were then determined and compared to the PFU produced by the 10^6 ^T4 sample (% PFU Remaining). For the 60 min panel, the phage and WT OMV preparations were incubated for 1 h with mid log-phase MK496 cells, PFU were determined, and compared to the PFU produced by the 10^6 ^T4 sample (% PFU Remaining). (n = 9) (**B**) 10^6 ^T4 phage were mixed with 1 μg purified WT OMVs, then immediately ("0" min), and at 5 min intervals thereafter, samples were taken and chloroform was added to disrupt the OMVs and allow reversibly bound phage to be released. The T4 activity in each sample was determined by PFU titration and compared to the PFU produced by 10^6 ^T4 (% PFU Remaining). (n = 6) (**C**) Negative stain electron micrograph of the T4-OMV complex (size bar = 50 nm).

To further test the hypothesis that OMVs can help in defense against outer membrane-acting stressors, we investigated whether OMVs could protect *E. coli *from infection by bacteriophage T4. Co-incubation of T4 and OMVs resulted in a dramatic reduction of active phage (by approximately 90%), as measured by a reduction in plaque forming units (PFUs) (Figure 5A). To characterize the putative interaction between the phage and OMV we used the differential chloroform resistance properties of free or reversibly-bound phage, and irreversibly-bound phage. Chloroform is commonly used in the preparations of T4-phage lysates, since it acts to physically disrupt the membrane of living bacteria to free the replicated phage from cells, as well as to kill the bacteria and stop phage production [35]. Reversibly-bound phage are chloroform resistant and will remain infective following treatment, whereas irreversibly-bound phage are unable to cause infection following chloroform treatment. Immediately after mixing T4 and OMVs, and at 5 min intervals thereafter, the mixtures were treated with chloroform to break apart the OMVs. Following a 30 min shaking incubation at 37°C, the preparations were titered (Figure 5B). We found that inactivation of T4 by the addition of OMVs occurred very quickly. At the initial time point, we already observed a 60% reduction in infectious phage. By 5 min, we saw an 80% reduction in the free phage, and by one hour, we saw further reduction, until only approximately 10% of the original phage activity remained. Based on the time-course of the reduction in the numbers of active T4 in the chloroform-treated OMV-phage mixture, we concluded that T4 are binding to the OMVs in a fast and irreversible manner.

In order to reveal the longer-term effects of the presence of OMVs on T4 infectivity in a microenvironment, we observed the infection and reproduction of the phage in the mixture following a 1 h incubation with the titer strain. After we co-incubated the T4 and OMVs, we added this mixture to growing cultures of the titer strain and incubated for 1 hour instead of only 5 min. This timepoint is sufficient to allow several cycles of infection and allowed us to observe whether the OMVs in the mixture have an affect beyond the initial inactivation. To use as a comparison, we first determined the amount of free phage (10^5^) that produced the equivalent PFUs to the amount of infectious phage in the mixture when it was incubated with the titer strain for only 5 min (Figure 5A, 5 min). Then we compared the amount of PFUs formed after a 60 min incubation of cells incubated with 10^5 ^T4 or with the mixture of T4 and OMVs. We found that the sample containing the mixture of T4 and OMVs contained fewer infectious phage as compared to both the original 10^6 ^T4 as well as the 10^5 ^free T4 samples (Figure 5A, 60 min). This suggests that the addition of OMVs to T4 significantly reduces the infectivity of T4 over several generations of phage infection.

Finally, we used electron microscopy to determine whether complexes between T4 and OMVs could be visualized. We found many complexes between T4 and OMV (an example is shown in Figure 5C), and in these cases, T4 was in a similar orientation as was observed between T4 and bacterial cell wall [36]. These data support the model that released OMVs and vesiculation may contribute to the innate bacterial defense against outer-membrane acting stressors.

## Discussion

Understanding how bacteria manage to survive in hostile environments has been an important step towards understanding bacterial defense and pathogenesis. As our understanding of the bacterial world has increased, so has our appreciation of the complexity of the constant interactions that occur between bacteria and their environment. These include the well-studied interactions that occur between a pathogen and the host environment, as well as the less-appreciated interactions that occur between bacteria and the general environment. With the current escalation in the variety of antimicrobial resistant bacteria, a particularly critical topic concerning bacterial-environment interactions is the bacterial response to the presence of an antibiotic. Many reports discuss the different pathways that allow microbes to adapt to antibiotics and achieve antimicrobial resistance (drug export, target modification, etc.) [1,37-40], but how bacteria survive the initial antibiotic assault is less well understood. Additionally, it is not well understood how bacteria respond to challenge with sub-lethal concentrations of antibiotics. These concentrations are relevant to this study because they are likely to be encountered clinically, by bacteria within biofilm communities (where therapeutic concentrations of antibiotics cannot easily penetrate and high OMV concentrations exist [6]) and during improper antibiotic dosing regimens, as well as in antibiotic-contaminated niches in the general environment.

In this study we show that OMVs represent an exported form of an inducible innate defense to sub-lethal concentrations of AMPs for both non-pathogenic and pathogenic *E. coli*. The concept that OMVs enable antibiotic resistance has been presented for β-lactam drugs in several studies demonstrating OMVs can carry active β-lactamase [41,42]. However, the idea that OMVs themselves can confer protection, without the need for an enzymatic resistance, has been less well studied, with only one report demonstrating that chlorhexadine can be adsorbed by OMVs in *P. gingivalis *[8]. The protection we observe is specific for outer membrane-targeting stressors, and we show that vesiculation is highly induced upon treatment with AMPs for which the OMVs are protective. Furthermore, as OMV protection can affect not only immediate survival, but also the acquisition of adaptive antibiotic resistance in a dose-dependent manner, it is important to consider the role of vesiculation as a short-term, low dose, antimicrobial defense mechanism that can affect long-term survival.

We observed that OMV-mediated defense against antimicrobials was limited to compounds that act at the outer membrane (AMPs). An association between OMVs and antibiotics was previously reported in a study demonstrating the trafficking of gentamicin within *P. aeruginosa *OMVs, and in this case is was presumed that the gentamicin reached the lumen of the OMV [43]. In the case of either polymyxin B or colistin interactions, OMVs likely confer protection via an adsorption mechanism. There have been no enzymatic mechanisms of resistance discovered to date [17,44], and thus it is highly unlikely that the OMVs convey enzymatic protection. Interactions between outer membrane LPS and AMPs have already been well documented [16], and our results further support this mechanism. Purified OMVs provided dose-dependent protection for polymyxin-treated cultures (Figure 1D, 3B), and the type of OMV LPS was paramount to OMV-mediated polymyxin protection, as OMVs from the polymyxin-resistant ETEC strain were not protective (Figure 3A). This suggests that OMVs present binding sites, acting as cellular decoys, for outer membrane-targeting stressors.

Whether bacteria can produce a protective concentration of OMVs in a physiological environment is a valid consideration. We propose that AMP-protective concentrations of OMVs are likely to be achieved in relevant settings for several reasons. First, a 10-fold increase in OMV concentration was sufficient for a K12 *E. coli *strain to gain significant protection (e.g. for the *yieM *mutant, Figure 1A, B). Therefore, the basal level of OMV production by untreated ETEC (which is approximately 10-fold higher than lab strains of *E. coli *[45]), is already sufficiently high to provide some intrinsic OMV-based AMP defense. Pathogenic strains generally make constitutively more OMVs than laboratory strains [45], so this likely holds for other species as well. Second, AMP treatment induced OMV production another 7-fold beyond the already high basal level for ETEC. Indeed, the high basal level coupled with induced OMV production could help explain the previously noted high intrinsic resistance of ETEC to polymyxin B and colistin [22]. Finally, in a natural setting, such as a colonized host tissue or biofilm, there is a gradient of antibiotic concentration [46,47] as well as high concentrations of OMVs [6]. Together, the induction of already high basal levels of OMV production and the concentration by the host microenvironments would be sufficient to yield short-term, OMV-mediated AMP protection.

We did note the incomplete (albeit 50%) protection of ETEC by the purified OMVs (Figure 3A, B). If enough OMVs were used, it is possible that we could have achieved 100% protection, however, we felt that concentrations exceeding those used in this study would be unreasonable. It should be further emphasized that the goal of an immediate, innate bacterial defense mechanism is to quickly impart an advantage, not necessarily to achieve 100% protection. In addition, OMV-dependent modulation of the adaptive response to polymyxin B (Figure 4) suggests that there is likely an optimal level of OMV induction in response to AMPs. The optimal amount would be sufficient to achieve immediate protection, and maintain a viable population, while being low enough to allow bacteria exposure to the AMPs so that adaptive resistance would still be stimulated in that population.

The observation that AMPs specifically induced vesiculation suggests that OMV formation is a regulated response by the bacteria. The induction pathway depends at least partially on the ability of the AMP to bind LPS since the polymyxin did not induce vesiculation in the ETEC-R strain (Figure 3D). Recently, Fernandez et al discovered a sensor system in *Pseudomonas aeruginosa *that is distinct from the PhoP-PhoQ or PmrA-PmrB two component systems and that is responsible for sensing the polymyxin B peptide in more physiological conditions [48]. This system, composed of ParR-ParS, is tied to activation of the *arnBCADTEF *LPS modification system [48]. The existence of the ParR-ParS system suggests that there could be a similar sensor that responds to binding of AMPs to LPS and modulates OMV production. We propose that such a response to AMPs is what could lead to physiologically protective levels of OMVs.

To extend our investigation of ubiquitous stressors found in both host and natural environments that attack via the outer membrane, we chose to investigate T4 bacteriophage [16,28]. T4 is a well-studied bacteriophage and is already linked to overproduction and release of outer membrane [31]. Our results show that there is significant binding and reduction of infection when T4 was pre-incubated with OMVs (Figure 5). In order to investigate the binding interaction between T4 and OMVs, we took advantage of the resistance of T4 to chloroform treatment. Chloroform disrupts the bacterial outer membrane and results in the release of active T4 only if the binding is reversible. T4 phage undergoes two general steps in binding prior to injection of its genetic material, the first is a reversible step where long tail fibers bind the LPS of the outer membrane of the host, the second is an irreversible step whereby the short tail fibers identify and bind to a cognate host factor [49]. Once this second step occurs, chloroform treatment will not free the phage to allow them to infect and replicate (visualized by the formation of plaques on a lawn of plated *E. coli*). Upon addition of OMVs, we clearly observed an immediate reduction in the population of infectious phage (Figure 5B), demonstrating that T4 binding to OMVs is quick and irreversible. Although we tried to amplify phage DNA from T4-OMV complexes, we could not definitively determine if the bound phage had injected its DNA into the OMV (data not shown).

When we compared the infectivity of T4 in a mixture with OMVs and that of 10^5 ^T4 using conditions that allow several cycles of infection, we found that over the long-term, infectivity of T4 in the OMV mixture was reduced (Figure 5A, 60 min panel). This experiment highlights the ability of OMVs to continue binding and inactivating T4 beyond the initial binding event and thereby greatly impact the rate of bacterial infection by phage in the environment.

Our results suggest a model in which vesiculation is an inducible "innate immune" mechanism for bacterial defense. In this model, a community of bacteria encounters an outer membrane-acting stressor. When the stressor is encountered, some bacteria will die, while vesiculation is induced for others. This is beneficial for several reasons: the stressor is shed, relieving the cell of the stress, and also the local and global concentration of OMVs significantly increases, benefiting itself as well as neighboring cells by their ability to neutralize cell surface-acting stressors. The period of hyper-vesiculation also can offer an opportunity for a greater number of cells to modify their outer membrane and generate resistance, such as altering their LPS structure so that it resists binding to AMPs [27,33]. Therefore, while OMVs are a short-term defense against low-doses of cell wall stressors, vesiculation can also contribute to long-term protective mechanisms that Gram-negative bacteria use to extend life in hostile environments.

## Conclusions

OMVs can adsorb outer membrane-acting compounds including antimicrobial peptides and T4 bacteriophage, resulting in their loss of efficacy. OMVs interact with AMPs in a dose dependent manner and their interaction can lead to the complete adsorption of antimicrobial activity. In the case of bacteriophage, OMVs not only irreversibly bind the phage, but they also greatly reduce their ability to infect once attached to the OMV. We further determined that OMVs production was significantly induced in response to AMPs. While it is possible for OMVs at sufficient concentrations to provide 100% protection, we find that it is much more likely that vesiculation is a short-term response that can be upregulated to neutralize low doses of stressors as a way to "buy time" until a more persistent, adaptive resistance mechanism is expressed. Our results are consistent with the idea that OMV production can act as a modulated defensive response to certain outer membrane-acting stressors.

## Methods

### Strains and cultures

*E. coli *strain ADA600 carrying a plasmid for kanamycin resistance (MK496) was used in this study (WT) [9], along with a hyper-vesiculating isogenic strain ADA600 Δ*yieM *(MK1248, made by P1 phage transduction from the Keio collection knockout strain [50]) which does not carry the plasmid but encodes kanamycin resistance within the gene disruption cassette. The presence of a plasmid did not affect vesicle production or growth of ADA600 (data not shown). ETEC was obtained from the ATCC (strain 43886, O25:K98:NM) [45]. Since ADA600 does not encode alkaline phosphatase, MK318 (BW25113, [50]) was used for the AP leakage assay. Vesiculation phenotypes, responses, and antibiotic sensitivities were equivalent in both ADA600 and BW25113 strains (data not shown). Polymyxin B-resistant ETEC was generated by growing ETEC in the presence of 3.5 μg/mL polymyxin B overnight, plating the surviving culture, and growing new cultures in the presence of 5 μg/mL polymyxin B. ETEC-R was subsequently determined to be resistant to 15 μg/mL of polymyxin B. T4 D+ bacteriophage was used in this study. Bacterial cultures were grown in Luria-Bertani (LB) broth (10 g/L Bactotryptone, 5 g/L yeast extract, 10 g/L NaCl) or on LB agar plates (LB with 15 g/L BactoAgar) supplemented with 50 μg/mL kanamycin (Sigma) or 5 μg/mL polymyxin B (Sigma) when appropriate. Overnight cultures (5 mL) were inoculated from individual colonies selected from an LB agar plate. All liquid cultures were grown using a shaking incubator (200 rpm) at 37°C.

Antimicrobials were purchased through Sigma Aldrich. Antibiotic stocks (polymyxin B, 2.5 mg/mL; colistin 1 mg/mL) and 10-fold dilutions of stocks were prepared fresh the day of each experiment, stored with constant agitation at 4°C, and used only the day they were prepared.

### Survival assay

Cultures of WT and mutant *E.coli *were grown in LB with kanamycin (50 μg/mL) at 37°C to an OD_600 _0.45. Antibiotics were added as indicated, treated and untreated cultures were incubated further (37°C, 2 h), then a portion of the culture plated at 10^-6^, 10^-7^, and 10^-8 ^dilutions on LB agar plates containing kanamycin, plates were grown for 16 h at 37°C, and colony forming units (CFU) were counted to determine CFU/mL. For ETEC cultures, no kanamycin was used.

### OMV purification and quantitation

OMVs were prepared from overnight cultures as described previously [9]. Briefly, cells were pelleted (10,000 *g*, 15 min, 4°C) and the resulting supernatants were filtered (0.45 μm, Millipore Durapore PVDF membrane). Filtrates were centrifuged (38,400 *g*, 3 h, 4°C) and the OMV containing pellets were resuspended in Dulbecco's phosphate buffered saline (0.8 g KCl, 0.8 g KH_2_PO_4_, 46.8 g NaCl, 4.6 g Na_2_HPO_4_, 0.4 g MgCl_2_*6H_2_O, 0.4 g CaCl_2 _in 4L dH_2_O) supplemented with 0.2 M NaCl (DPBSS) and filter sterilized (0.45 μm Ultra-free spin filters, Millipore). The total protein concentration in the purified OMV preparations was determined by Bradford Coomassie assay (Pierce), and the OMV concentrations used in subsequent assays refer to this protein-based value.

To quantitate OMV yield, broth cultures were inoculated at a 1:1000 dilution and grown in LB at 37°C until the culture reached an OD600 of 0.5-0.6 at which point it was either treated or not, as indicated, and grown overnight (16 h) at 37°C. At the time of vesicle harvest, a portion of the culture was plated on LB agar to determine CFU/mL. OMVs were isolated as described above. Two previously established methods, an outer membrane protein-based and lipid-based assay [9,51], were used to quantitate vesiculation in treated and untreated cultures. OMV pellets were boiled in Laemmli buffer and separated by SDS-PAGE. Gels were stained with SYPRO Ruby Red (Molecular Probes). Bands representing OmpF/C and OmpA were quantified by densitometry (NIH Image J software). Lipid in the OMV pellets was measured using the lipophilic dye FM4-64 (Invitrogen), as described previously [51]. In both cases, OMV production was normalized by dividing by the CFU/mL for each culture. Vesiculation measurements by both protein and lipid methods were very similar, therefore only protein values are shown. To determine relative OMV induction, OMV/CFU values for treated cultures were divided by OMV/CFU of an untreated culture.

### OMV-mediated protection assays

Cultures of WT *E. coli *were grown in LB at 37°C to OD_600 _0.45 and treated with indicated concentrations of antibiotics alone, with OMVs alone (5 μg/mL), or simultaneously with OMVs and antibiotics. Cultures were incubated (2 h, 37°C) and then plated on LB agar containing kanamycin to determine CFUs. For ETEC experiments, indicated concentrations of polymyxin B, and OMVs derived from ETEC, WT, and ETEC-R strains were used, and cultures were plated on LB agar without kanamycin. Relative growth (% Survival) was determined by dividing the CFUs obtained from treated cultures by the CFUs from cultures without antibiotic.

To titrate OMV-mediated protection, OMVs and antimicrobials were co-incubated in 5 mL LB (2 h, 37°C) at the indicated concentrations. The mixture was centrifuged (38,400 *g*, 1 h), and the supernatant (OMV-pretreated media) transferred to a new tube. Meanwhile, cultures of WT or ETEC *E. coli *(5 mL) were grown to OD_600 _0.45, centrifuged (4100 *g*, 10 min), and the media was removed. The cell pellets were then resuspended to their original culture volume (5 mL) with OMV-pretreated media, incubated (2 h, 200 rpm, 37°C), and dilution-plated on LB agar plates (containing kanamycin for WT, not ETEC, cultures) to determine CFU/mL. Relative growth (% Survival) was determined by dividing the CFUs obtained from treated cultures by the CFUs from cultures without antibiotic.

### Alkaline phosphatase cell integrity assay

*E. coli *MK318 cultures were treated for 2 h with 0.75 μg/mL polymyxin B, or 0.5 μg/mL colistin. A portion of the treated and untreated cultures was dilution-plated for CFU/mL determination. Following the treatment, cells were pelleted (4,100 *g*, 10 min, 4°C), and the supernatant was cleared of OMVs (38,400 g, 2 h, 4°C). AP was detected in 150 μL samples of OMV-free supernatant (S) and the whole cell pellets (WC) using the Anaspec Sensolyte pNPP alkaline phosphatase assay kit per the manufacturer's instructions. Briefly, 50 μl of sample was applied in duplicate to each well of a 96-well plate (Corning), then 50 μl of pNPP substrate solution was added. Absorbance at 405 nm was measured (Fluostar Optima) after 2 h. AP concentrations in samples were derived using a standard curve generated using known concentrations of AP. The ratios of AP in the OMV-free supernatant compared to the whole cells (S/WC) were then normalized to the CFU/mL in the original cultures.

### Polymyxin B resistance plate assay

To assess the time-course of the acquisition of adaptive polymyxin B resistance, the procedure described for the OMV protection assay was used, except that following the indicated treatment of the ETEC cultures with ETEC OMVs and polymyxin B, polymyxin B alone, or LB alone, cultures were streaked on LB agar and LB agar containing 5 μg/ml of polymyxin B with a sterile applicator at 1 h intervals for up to 7 h.

### T4 titration

T4 D+ phage titering was assessed using MK496 as the host strain. Several 10-fold dilutions of a high-concentration lysate were made, 100 μL of each of these dilutions was then combined with 100 μL of MK496 for 5 min, the 200 μL samples were then added to warmed (55°C) top agar (Bactotryptone 13 g/L, NaCl 8 g/L, Na-Citrate-2H_2_O 2 g/L, Glucose 3 g/L, and Bactoagar 6.5 g/L) that was diluted 1:2 with LB, and the top agar mixture was plated on T4 plates (Bactotryptone 13 g/L, NaCl 8 g/L, Na Citrate-2H_2_O 2 g/L, Glucose 1.3 g/L, and Bactoagar 10 g/L). T4 top agar and T4 plates were used for all titrations and experiments using phage. Plaque forming units (PFU) were counted by examining bacterial lawns following overnight incubation at 37°C.

### T4-OMV assays

10^6 ^T4 phage were co-incubated with 1 μg WT OMVs in 5 mL LB (2 h, 37°C). Following the incubation, 100 μL of the solution was mixed with 100 μL of a stationary phase culture of MK496 then mixed with 4 mL of a T4 top agar solution (3 mL T4 top agar, 1 mL LB) and after 5 min at 25°C, plated on a T4 plate. To determine the effect of OMVs on T4 chloroform resistance, 13 identical samples were prepared, each containing OMVs (1 μg) and 5 mL of LB media containing 10^6 ^T4. Chloroform (200 μL) (Mallinckrodt Chemical) was added to the first sample immediately upon gentle mixing, and to each of the other 12 samples at intervals every 5 min until 60 min. Following a 30 min incubation with the chloroform, at 37°C the samples were diluted and titered on MK496 to determine PFU as described above. The PFU titer of each sample was divided by the PFU produced by incubations with 10^6 ^T4 (% PFU Remaining).

For 60 minute incubations, MK496 cultures (5 mL) were grown to an OD_600 _of 0.5-0.6, centrifuged (4100 *g*, 10 min, 4°C), supernatant was removed, and pellets resuspended in the following 5 mL LB preparations using gentle pipetting: 10^6 ^T4 alone, 1 μg WT OMV alone, 10^5 ^T4 phage alone, or 10^6 ^T4 that had been preincubated with 1 μg WT OMV (2 h, 37°C). Cultures were allowed to grow for 1 h at 37°C, then diluted, if necessary. A portion (200 μL) of each sample was mixed with T4 top agar and plated as described above. As MK496 was already present in the samples, they did not need to be mixed with fresh cells for titration. The PFU of each sample was divided by the PFU resulting from the incubation with 10^6 ^T4 (% PFU Remaining).

### Electron microscopy

In advance, 400 mesh copper grids with carbon films deposited on them (Electron Microscopy Sciences, #CF400-cu) were cleaned via glow discharge for 1.5 min on a Harrick Plasma Cleaner (PDC-32G). Samples were prepared by applying 10 μL to the grid (10^3 ^T4 phage along with 0.001 μg WT OMVs in DPBSS) and incubated 2 min, grids were then washed with 5 drops of 1% aqueous uranyl acetate (Electron Microscopy Sciences). The last drop was left to incubate on the grid for 1.5 min before being wicked off by torn filter paper. Grids were left to dry for 5 min before being viewed on a Tecnai 12 by FEI with a 1024 × 1024 Gatan Multi-Scan Camera model 794.

### Statistics

Error bars throughout the figures refer to standard error for all experiments. Asterisks in figures indicate significance as measured by Student's T-test assuming equal variance: *p ≤ 0.05, **p ≤ 0.001, and ***p ≤ 0.0005, n ≥ 6; n values for each experiment are indicated in each figure legend. Each n is an independent experiment done in at least duplicate on different days.

## Authors' contributions

AJM conducted all experiments, was the primary person to develop all of the assays, and drafted the manuscript. MJK helped to conceive the study, participated in the experimental design and coordination, and helped to draft the manuscript. Both have given final approval to this work and have no conflicts of interest to report.

## Supplementary Material

Additional file 1 **Figure S1. Mass spectroscopic analysis of lipid A**. Lipid A was purified as described below from MK318 (**A**), MK496 (**B**), MK1248 (Δ*yieM *derivative of MK496) (**C**), ETEC (**D**), and ETEC-R (polymyxin B resistant derivative of ETEC) (**E**). Samples were applied to normal phase LC/MS and relevant areas of the spectrum are shown. Lipid A samples were prepared as described previously [52]. Normal phase liquid chromatography was performed on an Agilent 1200 Quaternary LC system equipped with an Ascentis Silica HPLC column, 5 μm, 25 cm × 2.1 mm (Sigma-Aldrich, St. Louis, MO). Mobile phase A consisted of chloroform/methanol/aqueous ammonium hydroxide (800:195:5, v/v); mobile phase B consisted of chloroform/methanol/water/aqueous ammonium hydroxide (600:340:50:5, v/v); mobile phase C consisted of chloroform/methanol/water/aqueous ammonium hydroxide (450:450:95:5, v/v). The elution scheme for the column after loading of the sample was as follows: 100% mobile phase A was held constant for 2 min, followed by a linear increase to 100% mobile phase B over 14 min. The column was then held at 100% mobile phase B for 11 min, followed by a linear change to 100% mobile phase C over 3 min. Finally, the mobile phase was set at 100% C for 3 min. The column was returned to 100% mobile phase A over the course of 0.5 min and then held at 100% mobile phase A for 5 min prior to application of the next sample. The LC flow rate was 300 μL/min. The post-column splitter diverted approximately 10% of the LC effluent into the mass spectrometer, a QSTAR XL quadrupole time-of-flight tandem mass spectrometer (Applied Biosystem, Foster City, CA). Instrumental settings for negative ion electrospray (ESI) and MS/MS analysis of lipid species were as follows: IS = -4500 V; CUR = 20 psi; GSI = 20 psi; DP = -55 V; and FP = -150 V. The MS/MS analysis used nitrogen as the collision the gas. Each injection consisted of about 0.1% of the total lipid extracted from a 20 mL *E. coli *culture, typically in 10 μL chloroform/methanol (2:1, v/v). Data analysis was performed using Analyst QS software (Applied Biosystem, Foster City, CA). (n = 3). Click here for file

Additional file 2 **Figure S2. Growth of untreated WT *E. coli *is unaffected by the addition of OMVs**. Relative survival (% Survival) of antibiotic-free cultures of mid-log WT *E. coli *cultures supplemented with 4 μg/mL OMVs (2 h, 37°C)(Untreated +OMV) compared with non-supplemented, antibiotic-free cultures (Untreated). (n = 9). Click here for file

Additional file 3 **Figure S3. Protein content in untreated and polymyxin B-treated culture fractions are similar**. Equivalent volumes of sub-cellular fractions from untreated (**A**) and 0.75 μg/mL polymyxin B-treated (2 h, 37°C) (**B**) log-phase cultures of MK496 were separated by SDS-PAGE and stained using SYPRO Ruby Red. Whole cell (WC), cytoplasm (C), inner membrane (IM), periplasm (PP), outer membrane (OM), and OMV fractions were isolated and purified using previously described methods [53]. The protein content and protein ratios in each fraction are very similar for both conditions. (n = 3). Click here for file
