# Supplementary figures and images for: Contribution of bacterial outer membrane vesicles to innate bacterial defense
Source: BMC Microbiol. 2011 Dec 1;11:258. doi: 10.1186/1471-2180-11-258 (PMC3248377; doi:10.1186/1471-2180-11-258)

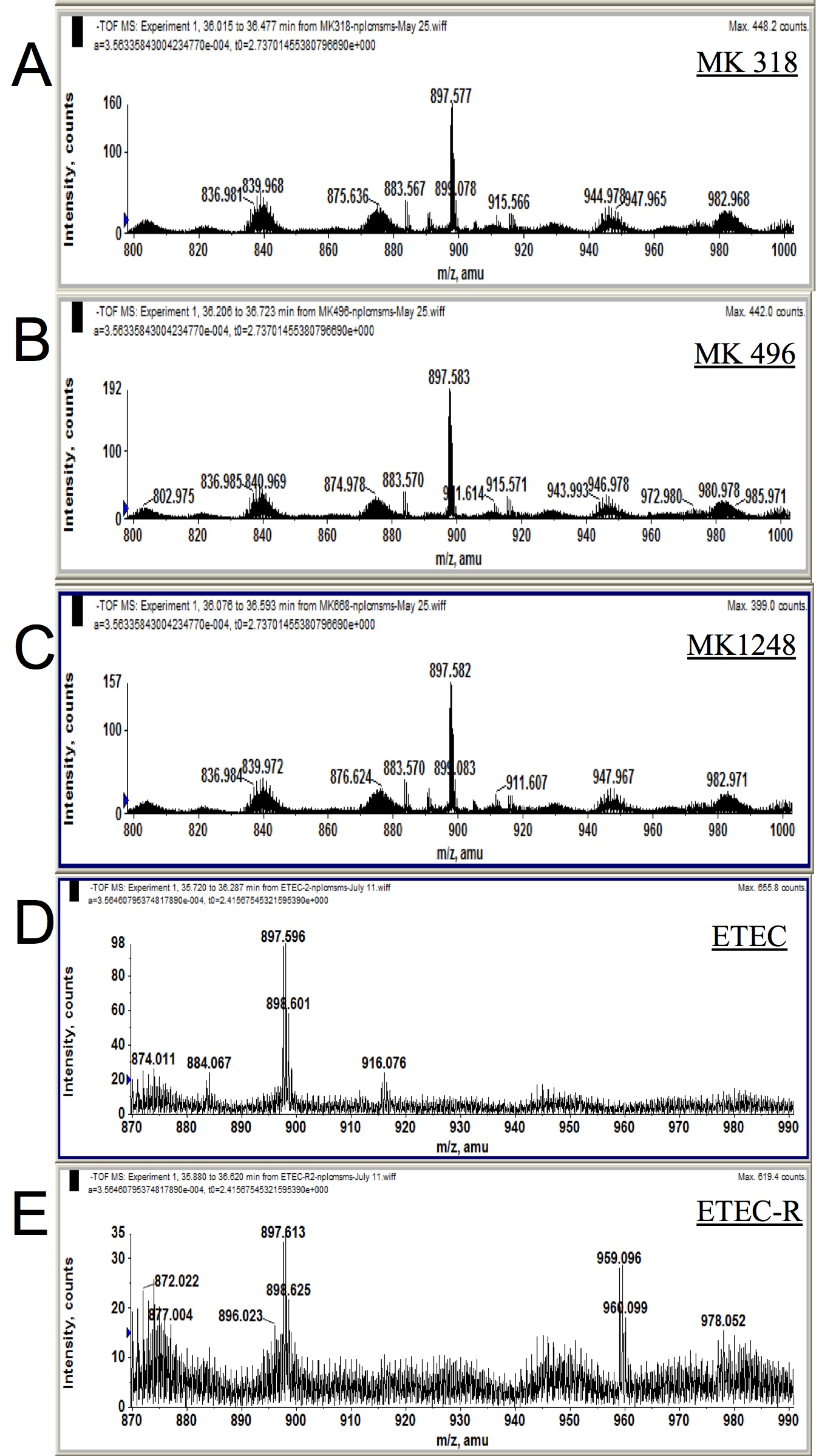

Supplement: Additional file 1 — Figure S1. Mass spectroscopic analysis of lipid A. Lipid A was purified as described below from MK318 (A), MK496 (B), MK1248 (ΔyieM derivative of MK496) (C), ETEC (D), and ETEC-R (polymyxin B resistant derivative of ETEC) (E). Samples were applied to normal phase LC/MS and relevant areas of the spectrum are shown. Lipid A samples were prepared as described previously [52]. Normal phase liquid chromatography was performed on an Agilent 1200 Quaternary LC system equipped with an Ascentis Silica HPLC column, 5 μm, 25 cm × 2.1 mm (Sigma-Aldrich, St. Louis, MO). Mobile phase A consisted of chloroform/methanol/aqueous ammonium hydroxide (800:195:5, v/v); mobile phase B consisted of chloroform/methanol/water/aqueous ammonium hydroxide (600:340:50:5, v/v); mobile phase C consisted of chloroform/methanol/water/aqueous ammonium hydroxide (450:450:95:5, v/v). The elution scheme for the column after loading of the sample was as follows: 100% mobile phase A was held constant for 2 min, followed by a linear increase to 100% mobile phase B over 14 min. The column was then held at 100% mobile phase B for 11 min, followed by a linear change to 100% mobile phase C over 3 min. Finally, the mobile phase was set at 100% C for 3 min. The column was returned to 100% mobile phase A over the course of 0.5 min and then held at 100% mobile phase A for 5 min prior to application of the next sample. The LC flow rate was 300 μL/min. The post-column splitter diverted approximately 10% of the LC effluent into the mass spectrometer, a QSTAR XL quadrupole time-of-flight tandem mass spectrometer (Applied Biosystem, Foster City, CA). Instrumental settings for negative ion electrospray (ESI) and MS/MS analysis of lipid species were as follows: IS = -4500 V; CUR = 20 psi; GSI = 20 psi; DP = -55 V; and FP = -150 V. The MS/MS analysis used nitrogen as the collision the gas. Each injection consisted of about 0.1% of the total lipid extracted from a 20 mL E. coli culture, typically in 10 μL chloroform/metha [file 1471-2180-11-258-S1.JPEG]

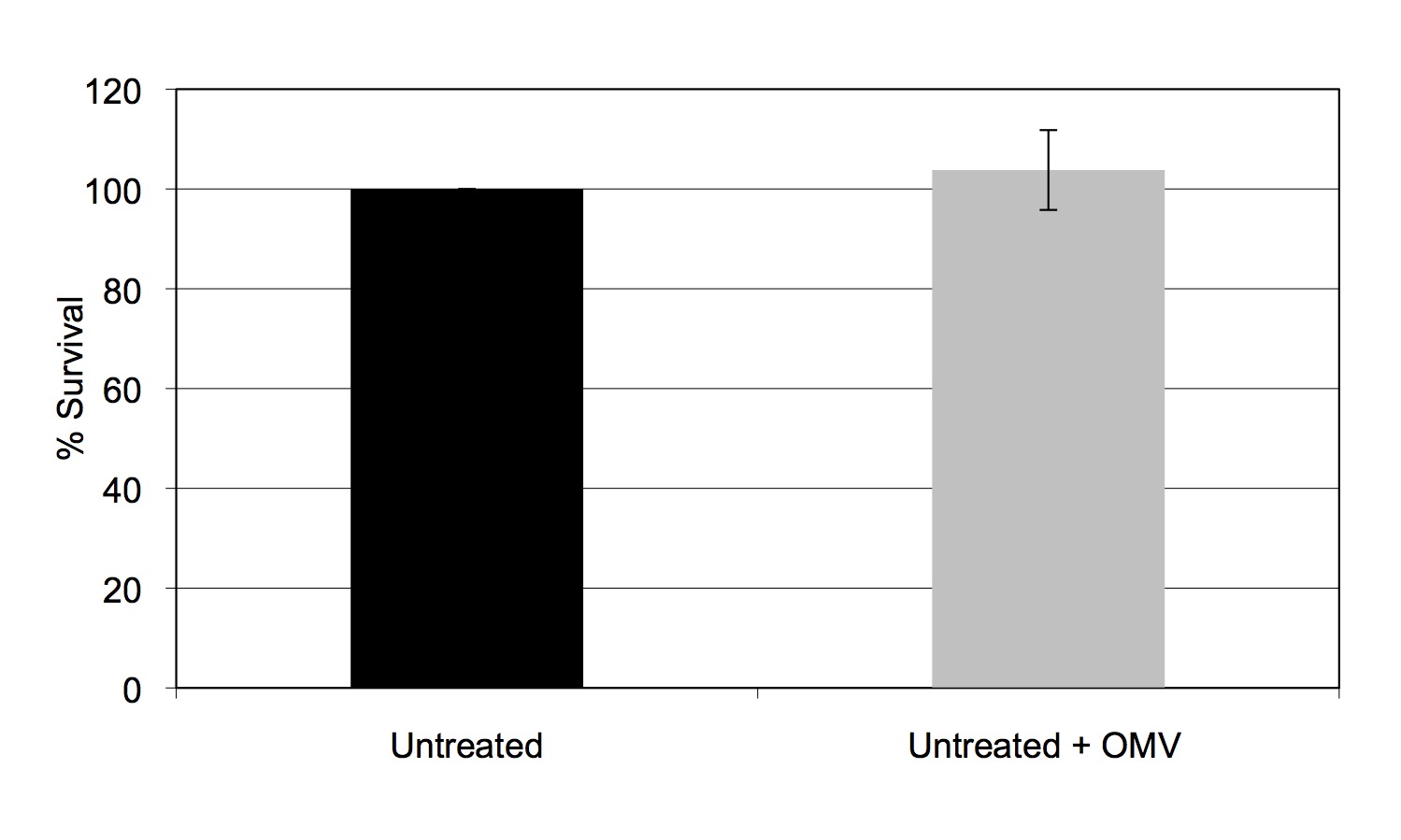

Supplement: Additional file 2 — Figure S2. Growth of untreated WT E. coli is unaffected by the addition of OMVs. Relative survival (% Survival) of antibiotic-free cultures of mid-log WT E. coli cultures supplemented with 4 μg/mL OMVs (2 h, 37°C)(Untreated +OMV) compared with non-supplemented, antibiotic-free cultures (Untreated). (n = 9). [file 1471-2180-11-258-S2.JPEG]

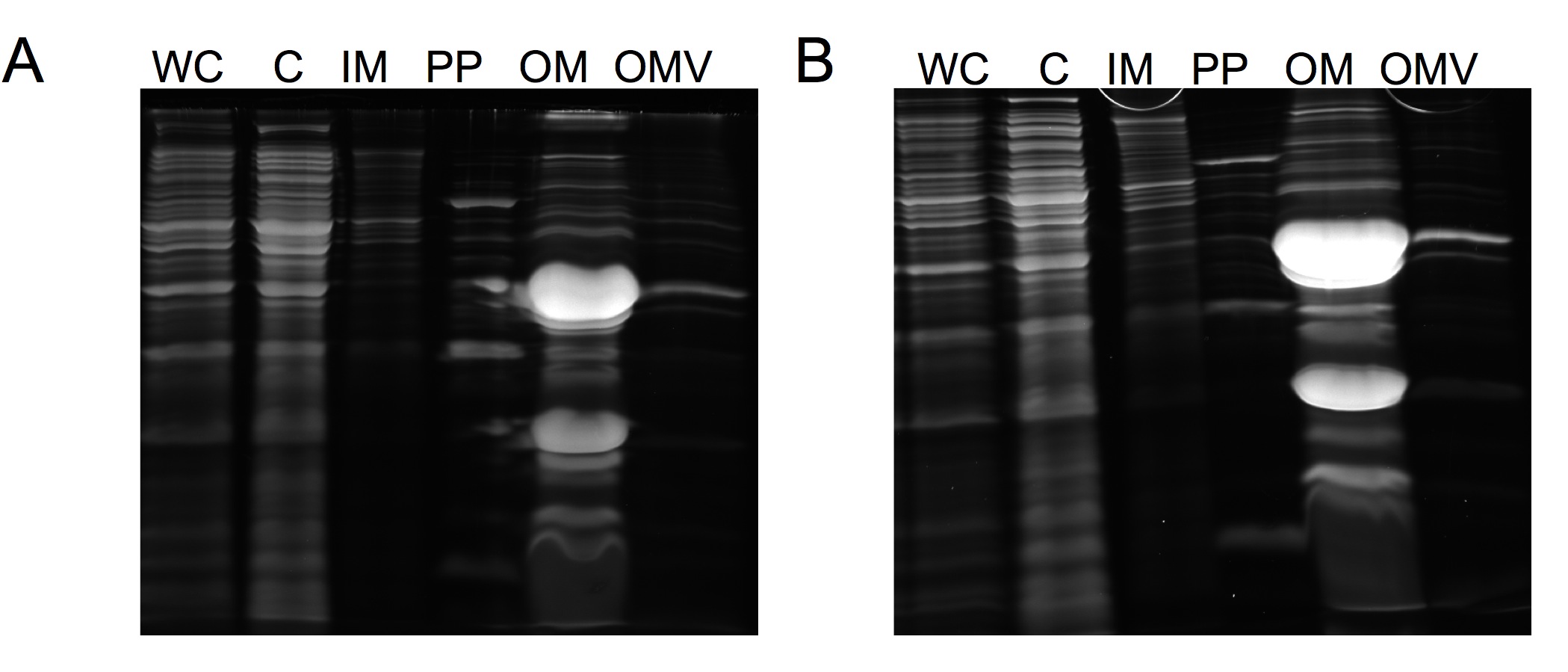

Supplement: Additional file 3 — Figure S3. Protein content in untreated and polymyxin B-treated culture fractions are similar. Equivalent volumes of sub-cellular fractions from untreated (A) and 0.75 μg/mL polymyxin B-treated (2 h, 37°C) (B) log-phase cultures of MK496 were separated by SDS-PAGE and stained using SYPRO Ruby Red. Whole cell (WC), cytoplasm (C), inner membrane (IM), periplasm (PP), outer membrane (OM), and OMV fractions were isolated and purified using previously described methods [53]. The protein content and protein ratios in each fraction are very similar for both conditions. (n = 3). [file 1471-2180-11-258-S3.JPEG]
